# Early neurological deterioration in acute ischemic stroke patients after intravenous thrombolysis with alteplase predicts poor 3-month functional prognosis - data from the Thrombolysis Implementation and Monitor of Acute Ischemic Stroke in China (TIMS-China)

**DOI:** 10.1186/s12883-022-02737-8

**Published:** 2022-06-07

**Authors:** Fengli Che, Anxin Wang, Yi Ju, Yarong Ding, Honglian Duan, Xiaokun Geng, Xingquan Zhao, Yongjun Wang

**Affiliations:** 1 Present Address: Department of Neurology, Capital Medical University, Beijing, China; 2 Department of Neurology, Capital Medical University, Beijing, China; 3 Tiantan Neuroimaging Center for Excellence, Beijing Tiantan Hospital, Capital Medical University, Beijing, China; 4 Research Unit of Artificial Intelligence in Cerebrovascular Disease, Chinese Academy of Medical Sciences, Beijing, China; 5 China National Clinical Research Center for Neurological Diseases, Advanced Innovation Center for Human Brain Protection, Beijing Tiantan Hospital, Capital Medical University, Beijing, China

**Keywords:** Acute ischemic stroke, Intravenous thrombolysis, Early neurological deterioration, Prognosis

## Abstract

**Background:**

We aimed to investigate the risk factors of early neurological deterioration (END) after intravenous thrombolysis with recombinant tissue-type plasminogen activator (rt-PA) and the relationship between END and poor 3-month functional outcomes.

**Methods:**

Patients who accepted intravenous recombinant rt-PA were enrolled continuously. END was defined as an increase in National Institute of Health Stroke (NIHSS) score ≥ 4 points or death within 24 hours after intravenous thrombolysis. The modified Rankin Scale (mRS) score was recorded to evaluate the functional outcome of stroke, and the poor 3-month prognosis was defined as an mRS score ≥ of 3. Univariate and multivariate analyses were used to analyze the risk factors of END. The relation between END and 3-month functional outcome was analyzed by multivariate logistic regression analysis.

**Results:**

A total of 1107 patients (mean age, 63.42 ± 11.33 years; 673 males) were included in the final analysis, and 81(7.32%) patients had END. In multivariate analysis, the serum glucose level was significantly associated with END; the odds ratio was 1.10 (95% CI 1.03 to 1.18, *p* = 0.004). The multivariate logistic analysis showed END has a notable association with the poor 3-month functional recovery even after adjusting for confounding factors; the adjusted OR was 8.25 (95% CI 3.77 to 18.03, *p* < 0.0001).

**Conclusions:**

The initial serum glucose level might be an independent risk factor of END, and END might predict a poor 3-month prognosis.

**Supplementary Information:**

The online version contains supplementary material available at 10.1186/s12883-022-02737-8.

## Introduction

Stroke has become one of the leading causes of death and disability in humans, and there has been a high incidence of stroke in China [1, 2]. It has been confirmed that target-vessel revascularization is the most effective method to reduce the disability and mortality of patients. Meanwhile, intravenous thrombolysis with recombinant tissue-type plasminogen activator (rt-PA) has been the most economical and convenient treatment [3]. However, some studies have found that some patients still suffered severe neurological deterioration after receiving intravenous thrombolysis, which resulted in prolonged hospitalization and severe adverse prognosis [4]. This study explored the risk factors of early neurological deterioration (END) and the correlation between END and 3-month functional prognosis.

## Methods

### Study population

The data were obtained from the Thrombolysis Implementation and Monitor of Acute Ischemic Stroke in China (TIMS-CHINA) database - a multicenter prospective stroke registry program that enrolled patients who received intravenous tPA within 4.5 hours after symptom onset from May 2007 to July 2012 in China [5]. Some previous pieces of literature have reported the trial design and some results of the study [6, 7]. The ethics committee approved the study protocol of Beijing Tiantan Hospital with the Helsinki Declaration. The quality monitoring committee of TIMS-China and the Contract Research Organization independently have been regularly monitoring the registry. All participants had signed written consent.

### Definition of END and clinical outcome measurement

END was defined as an increase of NIHSS (National Institute of Health Stroke) score ≥ 4 points or death within 24 hours after intravenous thrombolysis [8]. The primary outcome was poor 3-month functional recovery, expressed as a modified Rankin Scale (mRS) score ≥ 3. The secondary outcomes were sICH (symptomatic intracranial hemorrhage) [9] and mortality at 7 days and 90 days. We used the definitions of sICH in the following three studies: Safe Implementation of Thrombolysis in Stroke-Monitoring Study (SITS-MOST) [8], National Institute of Neurological Disorders and Stroke (NINDS) [10], and European Cooperative Acute Stroke Study II (ECASS II) [11].

### Statistical analysis

Continuous variables were described by means (standard deviations [SDs]) or medians (interquartile ranges [IQRs]). Categorical variables were presented as frequencies and percentages. The baseline characteristics of patients between the END group and the non-END group were compared by Wilcoxon rank-sum tests for continuous variables and X^2^ test for categorical variables. Univariate and multivariate logistic regression was used to estimate the odds ratios (ORs), the corresponding 95% confidence intervals (CIs), and the adjusted ORs with their 95% CI. The multiple ordinal regression was used to test the distribution of mRS at 3-month of patients. SAS software performed all statistical analyses, version 9.4 (SAS Institute Inc., Cary, NC, USA). All *P* values were two-sided, with *P* < 0.05 considered statistically significant.

## Results

### Baseline characteristics

**Fig. 1:**
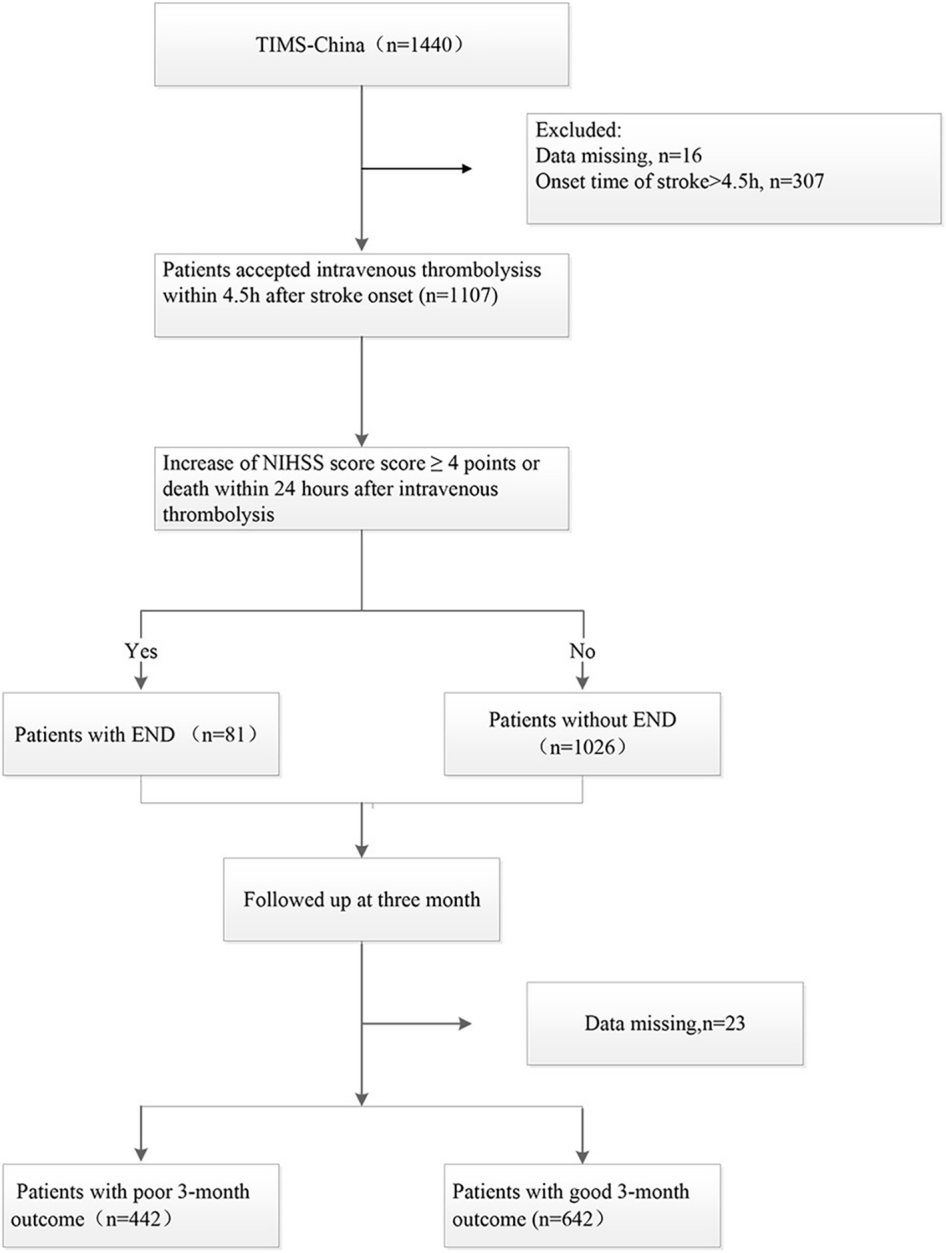
Flow chart of eligible patients. END indicates early neurological deterioration. NIHSS, National Institute of Health Stroke; mRS, modified Rankin Scale; TIMS-China, Thrombolysis Implementation and Monitor of Acute Ischemic Stroke in China

A total of 1107 consecutive patients (mean age, 63.42 ± 11.33 years; 673 males) were included in the final analysis, among which 81(7.32%) patients occurred END (Fig. 1). Between the END group and the non-END group, there were statistical differences in the history of prior stroke/TIA (2.47% vs.9.75%, *p* = 0.029), initial serum glucose level (9.00 ± 4.35 mmol/L vs.7.58 ± 2.87 mmol/L, *p* = 0.001), fibrinogen (3.47 ± 1.24 g/L vs. 3.23 ± 1.23 g/L, *p* = 0.040), low-density lipoprotein (3.20 ± 0.90 mmol/L vs. 2.92 ± 0.96 mmol/L, *p* = 0.003), cholesterol (5.08 ± 1.16 mmol/L vs. 4.85 ± 1.20 mmol/L, *p* = 0.021), SBP (systolic blood pressure) on admission (152.30 ± 18.56 mmHg vs. 147.57 ± 21.09 mmHg, *p* = 0.039), DBP (diastolic blood pressure) on admission (88.35 ± 11.86 mmHg vs. 85.70 ± 12.68 mmHg, *p* = 0.038), taking aspirin within 7 days before thrombolysis (38.27% vs. 65.20, *p* < 0.0001), and taking clopidogrel within 7 days before thrombolysis (13.58% vs. 23.59%, p = 0.039). There was no significant statistical difference in the neurological deficit on admission between the two groups. Concerning TOAST types, although the proportion of CE (cardioembolism) in the END group was higher than in the non-END group (28.21% vs. 18.69%), there was no difference in the etiology distribution between the two groups. In multivariate analysis, END has a significant correlation with the initial serum glucose level (OR,1.10, 95% CI 1.03–1.18; *p* = 0.004), taking aspirin within 7 days before thrombolysis (OR, 0.25, 95% CI 0.14–0.44; *p* < 0.0001), and taking clopidogrel within 7 days before thrombolysis (OR,0.39, 95% CI 0.19–0.82; *p* = 0.013). The demographics and clinical characteristics at the baseline of subjects in this study were demonstrated in Table 1, and multivariate logistic regression analysis for risk factors of END was shown in Table 2.

**Table 1:** Demographic and clinical characteristics in patients

Variables	END (***n*** = 81)	non-END (***n*** = 1026)	***P***-Value
Gender, male, n (%)	49(60.49)	624(60.82)	0.954
Age, mean ± SD (years)	64.47 ± 9.34	63.34 ± 11.48	0.636
Hypertension, n (%)	54(66.67)	598(58.34)	0.143
Diabetes mellitus, n (%)	17(20.99)	174(16.96)	0.356
Atrial fibrillation, n (%)	15(18.52)	182(17.74)	0.860
Hyperlipidemia, n (%)	3(3.70)	69(6.73)	0.288
prior stroke/TIA, n (%)	2(2.47)	100(9.75)	0.029
Current smoking, n (%)	20(24.69)	361(35.19)	0.056
Glucose, mean (SD), mmol/L	9.00 ± 4.35	7.58 ± 2.87	0.001
WBC, mean (SD), × 10^9^/L	8.32 ± 2.85	7.87 ± 2.70	0.216
FBG, mean (SD), g/L	3.47 ± 1.24	3.23 ± 1.23	0.040
LDL, mean (SD), mmol/L	3.20 ± 0.90	2.92 ± 0.96	0.003
TC, mean (SD), mmol/L	5.08 ± 1.16	4.85 ± 1.20	0.021
SBP on admission, mean (SD), mmHg	152.30 ± 18.56	147.57 ± 21.09	0.039
DBP on admission, mean (SD), mmHg	88.35 ± 11.86	85.70 ± 12.68	0.038
SBP at 2 hours after thrombolysis, mean (SD), mmHg	150.05 ± 22.31	143.03 ± 19.80	0.002
DBP at 2 hours after thrombolysis, mean (SD), mmHg	87.19 ± 14.28	83.42 ± 12.38	0.015
NIHSS on admission, IQR	12(3–28)	11(0–40)	0.559
Pre-admission mRS 0–2, n (%)	80(100)	1002(92.75)	0.398
Baseline medication within 7 days before thrombolysis			
Taking aspirin, n (%)	31(38.27)	669(65.20)	< 0.0001
Taking clopidogrel, n (%)	11(13.58)	242(23.59)	0.039
Taking other antiplatelets, n (%)	11(13.58)	156(15.20)	0.694
Taking hypoglycemic drugs, n (%)	0(0.00)	9(0.88)	1.000
Median door-to-needle time, IQR, h	1.82 ± 0.95	2.60 ± 9.90	0.971
TOAST subtypes			0.080
LAA, n (%)	43(55.13)	553(54.11)	
SAO, n (%)	4(5.13)	113(11.06)	
CE, n (%)	22(28.21)	191(18.69)	
Other, n (%)	9(11.54)	165(16.14)	

Abbreviations: *TIA* transient ischemic attack, *SBP* systolic blood pressure, *DBP* diastolic blood pressure, *WBC* white blood cell, *PLT* platelet, *INR* international normalized ratio, *PT* prothrombin time, *APTT* activated partial thromboplastin time, *FBG* fibrinogen, *NIHSS* National Institute of Health Stroke, *LDL* low-density lipoprotein, *TC* cholesterol, *IQR* interquartile ranges, *TOAST* Trial of Org 10,172 in Acute Stroke Treatment, *LAA* large-artery atherosclerosis, *Scale* SAO, small-artery occlusion, *CE* cardioembolism

**Table 2:** Multivariate logistic regression analysis for risk factors of END

Variables	OR	95% CI	*P*-value
prior stroke/TIA	3.14	0.74–13.38	0.123
Glucose	1.10	1.03–1.18	**0.004**
FBG	1.07	0.91–1.26	0.426
LDL	1.24	0.79–1.95	0.361
TC	0.93	0.66–1.32	0.685
SBP on admission	1.00	0.98–1.02	0.748
DBP on admission	1.01	0.98–1.04	0.555
SBP at 2 hours after thrombolysis	1.02	1.00–1.04	0.107
DBP at 2 hours after thrombolysis	1.00	0.97–1.03	0.960
Taking aspirin within 7 days before thrombolysis	0.25	0.14–0.44	**< 0.0001**
Taking clopidogrel used within 7 days before thrombolysis	0.39	0.19–0.82	**0.013**

Abbreviations: *OR* odds ratio, *CI* confidence interval, *TIA* transient ischemic attack, *SBP* systolic blood pressure, *DBP* diastolic blood pressure, *FBG* fibrinogen, *LDL* low-density lipoprotein, *TC* cholesterol

### Clinical outcomes

**Fig. 2:**
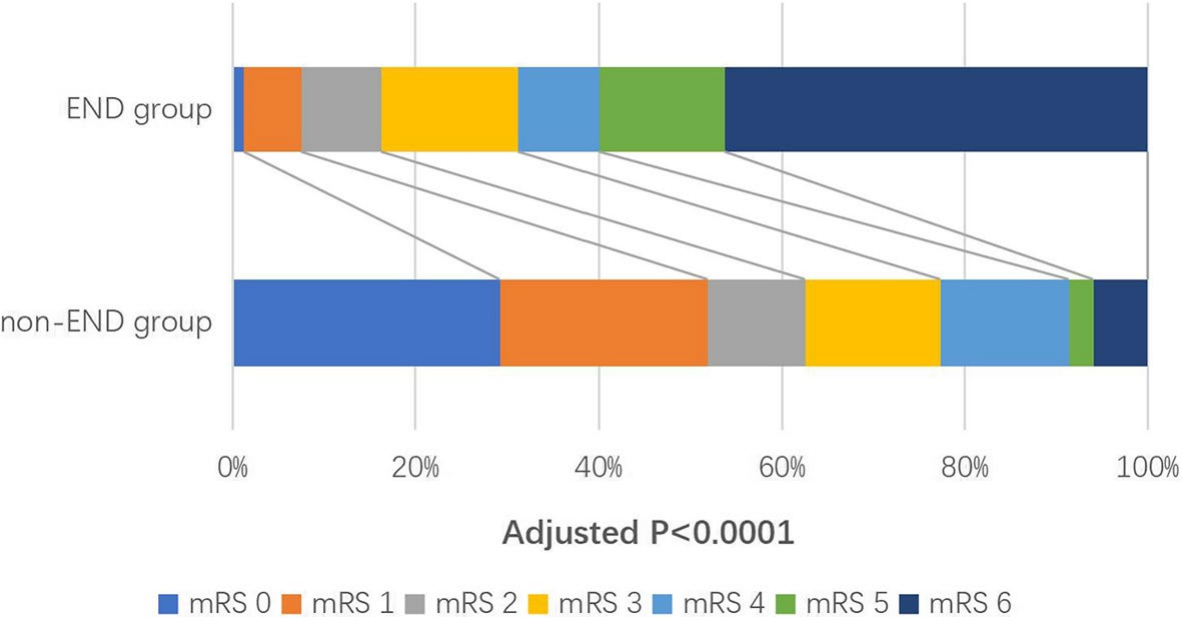
Distribution of modified Rankin scale (mRS) score after intravenous thrombolysis in patients with acute ischemic stroke

During the follow-up, 23 patients were excluded because of missing, and there were 1084 patients (97.92%) who had a 3-month mRS score. The proportion of poor function outcomes is 83.54% in the END group and 37.41% in the non-END group (crude OR 8.49; 95%CI 4.62 to 15.60; *P* < 0.0001). After adjusting the baseline variables as the history of prior stroke/TIA, initial serum glucose level, fibrinogen, low-density lipoprotein, cholesterol, SBP on admission, DBP on admission, taking aspirin within 7 days before thrombolysis, taking clopidogrel within 7 days before thrombolysis, and TOAST types, END has a statistical correlation with poor 3-month functional outcomes, the adjusted OR was 8.25(95% CI 3.77–18.03; *P* < 0.0001; Table 3). There was a numerical difference in the distribution of 3-month mRS among patients in the two groups (crude *p* < 0.0001), and this difference was still significant after adjusting confound factors (adjusted OR 11.74, 95%CI 7.58 to 18.18; *P* < 0.0001; Fig. 2). Regarding the secondary outcomes, END has a prominent correlation with SICH.

**Table 3:** Outcomes after intravenous thrombolysis in END group versus non-END group

Outcomes	No. (%) of patients		Unadjusted OR (95% CI)	***P*** value	Adjusted OR (95% CI)^a^	***P*** value
	END group (***n*** = 81)	non-END group (***n*** = 1026)				
Primary outcome						
mRS 3–6 at 3 months	66(83.54)	376(37.41)	8.49(4.62–15.60)	< 0.0001	8.25(3.77–18.03)	< 0.0001
Safety outcomes						
SICH (SITS-MOST)	15(18.52)	1(0.10)	232.83(−)	< 0.0001	109.77(−)	0.001
SICH (ECASS II)	30(37.04)	5(0.49)	120.12(44.74–322.50)	< 0.0001	90.46(19.84–412.44)	< 0.0001
SICH (NINDS)	30(37.04)	22(2.14)	26.84(14.47–49.79)	< 0.0001	12.53(5.15–30.49)	< 0.0001
Mortality at 7	26(32.50)	16(1.56)	30.39(15.39–60.01)	< 0.0001	20.92(7.45–58.72)	< 0.0001
Mortality at 3 months	36(45.00)	59(5.86)	13.13(7.86–21.94)	< 0.0001	8.06(3.91–16.62)	< 0.0001

^a^Adjusted baseline variables: prior stroke/TIA, Systolic BP before thrombolysis, Diastolic BP before thrombolysis, glucose before thrombolysis, FBG before thrombolysis, Systolic BP at 2 hours after thrombolysis, Diastolic BP at 2 hours after thrombolysis, LDL after thrombolysis, TC after thrombolysis, Aspirin use within 7 days after thrombolysis, clopidogrel use within 7 days after thrombolysis, OCSP subtypes

Abbreviations: *mRS* modified Rankin Scale, *SICH* symptomatic intracranial hemorrhage, *SITS-MOST* safe implementation of treatments in stroke-monitoring, *ECASS II* second European–Australasian acute stroke study, *NINDS* National Institute of Neurological Disorders and Stroke

And the adjusted OR (END with SICH defined by NINDS) was 12.53 (95% CI 5.15–30.49; *P* < 0.0001; Table 3). Meanwhile, END has a significant correlation with mortality at 7 days and mortality at 90 days, the adjusted OR was 20.92(95% CI 7.45–58.72; *P* < 0.0001; Table 3) and 8.06(95% CI 3.91–16.62; *P* < 0.0001; Table 3).

## Discussion

Our study aimed to explore the risk factors of END and the relationship between END and poor 3-month functional outcomes. In some studies, the incidence of END was significantly different due to the lack of a unified definition of END, which was from 5.8 to 34.9% [12–15]. Most studies have defined END as an increasing NIHSS score ≥ 4 points or death within 24 hours after intravenous thrombolysis, which was our study’s exact definition of END [8, 16]. Our study enrolled 1107 patients accepting intravenous thrombolysis, and 81 (7.32%) patients occurred END. Simonsen et al. studied 569 patients who received reperfusion therapy and found the incidence of END was 5.8% [14].

Concerning the risk factors of END, although some experts generally believe that elderly patients are more prone to END, it has not been confirmed in some studies [10, 15]. Some studies also have demonstrated the predictors of END as follows: diabetes [17, 18], neurological functional deficits on admission [19], and systolic BP [20–22].

There was no statistical difference in age between the END and non- END groups. Still, there were statistical differences in the history of prior stroke/TIA, initial serum glucose level, fibrinogen, low-density lipoprotein, cholesterol, SBP on admission, and DBP on admission between the two groups. However, in the multivariate analysis, our study found the initial serum glucose level was an independent risk factor of END; the odds ratio was 1.10 (95%CI 1.03 to 1.18, *p* = 0.004).

Our study found that antiplatelet therapy before thrombolysis might be the protective factor for END. The incidence of END in patients taking aspirin could be 0.25 times lower than in patients without aspirin, similar to patients taking clopidogrel within 7 days. Due to the limitation of our research, we had no further study on this result, and it would be a meaningful research focus in the future.

The series of pathophysiological reactions of the brain after intravenous thrombolysis, such as intracranial hemorrhage (ICH) [23, 24], malignant edema [25], early recurrent ischemic stroke [12], and early seizures [26], resulted in the aggravation of neurological deficit [20, 21]. Meanwhile, SICH had been the leading cause of END. Our study confirmed that the patients in the END group had a higher incidence of ICH than in the non-END group. The incidence of SICH (NINDS) in the END group patients could be 12.53 times higher than patients in the non-END group. Furthermore, END was significantly correlated with mortality at 7 days and mortality at 3 months. Compared the patients with non- END, the mortality at 7 days of patients with END could be 20.92 times higher, and the mortality at 3 months could be 8.06 times higher.

Our study still has some shortcomings. Firstly, our study did not analyze patients separately in anterior circulation stroke (ACS) and posterior circulation stroke (PCS) groups, which has been confirmed there was no significant difference in the incidence of END between ACS and PCS groups in a recent study [27]. Secondly, we also did not perform subgroup analysis based on the time of ICH, although we have realized that patients usually experience hemorrhagic transformation within 24 h after thrombolysis [28]. Thirdly, owing to the lack of records on other complications(i.e., hyperperfusion syndrome, early stroke recurrence) after intravenous thrombolysis, we were still unable to explain the pathological mechanism of END. Fourthly, confounding factors (i.e., mechanical ventilation, fluid resuscitation, decompressive craniectomy) were not eliminated in our study, which may lead to data bias. We look forward to furthering research in the future.

## Conclusions

Early neurological deterioration has a high incidence after intravenous thrombolysis, and the initial serum glucose level might be an independent risk factor of END. END might predict a poor 3-month prognosis. It might be essential to understand the underlying mechanism of END.

## Supplementary Information

**Additional file 1.**

## Data Availability

The datasets used and/or analyzed during the current study are available from the corresponding author on reasonable request.

## Abbreviations

TIA: Transient ischemic attack
SBP: Systolic blood pressure
DBP: Diastolic blood pressure
WBC: White blood cell
PLT: Platelet
INR: International normalized ratio
PT: Prothrombin time
APTT: Activated partial thromboplastin time
FBG: Fibrinogen
NIHSS: National Institute of Health Stroke
LDL: Low-density lipoprotein
TC: Cholesterol
IQR: Interquartile ranges
TOAST: Trial of Org 10,172 in Acute Stroke Treatment
LAA: Large-artery atherosclerosis
Scale: SAO, small-artery occlusion
CE: Cardioembolism
OR: Odds ratio
CI: Confidence interval
TIA: Transient ischemic attack
SBP: Systolic blood pressure
DBP: Diastolic blood pressure
FBG: Fibrinogen
LDL: Low-density lipoprotein
TC: Cholesterol
mRS: modified Rankin Scale
SICH: Symptomatic intracranial hemorrhage
SITS-MOST: Safe implementation of treatments in stroke-monitoring
ECASS II: Second European–Australasian acute stroke study
NINDS: National Institute of Neurological Disorders and Stroke

## References

[1] GBD 2016 Causes of Death Collaborators. Global, regional, and national age-sex specific mortality for 264 causes of death, 1980-2016: a systematic analysis for the global burden of disease study 2016. Lancet. 2017;390(10100):1151–210. 10.1016/S0140-6736(17)32152-9 PMC5605883 28919116

[2] Wu S, Wu B, Liu M, Chen Z, Wang W, Anderson CS, Sandercock P, Wang Y, Huang Y, Cui L (2019). Stroke in China: advances and challenges in epidemiology, prevention, and management. Lancet Neurol.

[3] Marko M, Posekany A, Szabo S, Scharer S, Kiechl S, Knoflach M, Serles W, Ferrari J, Lang W, Sommer P (2020). Trends of r-tPA (recombinant tissue-type plasminogen activator) treatment and treatment-influencing factors in acute ischemic stroke. STROKE.

[4] Thiebaut AM, Gauberti M, Ali C, Martinez De Lizarrondo S, Vivien D, Yepes M, Roussel BD (2018). The role of plasminogen activators in stroke treatment: fibrinolysis and beyond. Lancet Neurol.

[5] Liao XL, Wang CX, Wang YL, Wang CJ, Zhao XQ, Zhang LQ, Liu LP, Pan YS, Wang YJ (2013). Implementation and outcome of thrombolysis with alteplase 3 to 4.5 h after acute stroke in Chinese patients. CNS Neurosci Ther.

[6] Liao X, Wang Y, Pan Y, Wang C, Zhao X, Wang DZ, Wang C, Liu L, Wang Y (2014). Standard-dose intravenous tissue-type plasminogen activator for stroke is better than low doses. STROKE.

[7] Wang C, Yang Y, Pan Y, Liao X, Huo X, Miao Z, Wang Y, Wang Y (2018). Validation of the simplified stroke-thrombolytic predictive instrument to predict functional outcomes in Chinese patients. STROKE.

[8] Wahlgren N, Ahmed N, Dávalos A, Ford GA, Grond M, Hacke W, Hennerici MG, Kaste M, Kuelkens S, Larrue V (2007). Thrombolysis with alteplase for acute ischaemic stroke in the safe implementation of thrombolysis in stroke-monitoring study (SITS-MOST): an observational study. LANCET.

[9] Zhou H, Chen W, Pan Y, Suo Y, Meng X, Li H, Wang Y (2021). Effect of sex differences on prognosis of intravenous thrombolysis: data from the thrombolysis implementation and monitor of acute ischemic stroke in China (TIMS-China). Stroke Vasc Neurol.

[10] Mione G, Ducrocq X, Thilly N, Lacour JC, Vespignani H, Richard S (2016). Outcome of intravenous recombinant tissue plasminogen activator for acute ischemic stroke in patients aged over 80 years. Geriatr Gerontol Int.

[11] Larrue V, von Kummer RR, Müller A, Bluhmki E (2001). Risk factors for severe hemorrhagic transformation in ischemic stroke patients treated with recombinant tissue plasminogen activator: a secondary analysis of the European-Australasian acute stroke study (ECASS II). STROKE.

[12] Awadh M, MacDougall N, Santosh C, Teasdale E, Baird T, Muir KW (2010). Early recurrent ischemic stroke complicating intravenous thrombolysis for stroke: incidence and association with atrial fibrillation. STROKE.

[13] Nanri Y, Yakushiji Y, Hara M, Eriguchi M, Okada R, Yukitake M, Hara H (2013). Stroke scale items associated with neurologic deterioration within 24 hours after recombinant tissue plasminogen activator therapy. J Stroke Cerebrovasc Dis.

[14] Simonsen CZ, Schmitz ML, Madsen MH, Mikkelsen IK, Chandra RV, Leslie-Mazwi T, Andersen G (2016). Early neurological deterioration after thrombolysis: clinical and imaging predictors. Int J Stroke.

[15] Huang ZX, Huang Y, Zeng J, Hao H, Petroski GF, Lu H, Liu X, Liu Z (2020). Admission glucose levels may increase the risk for early neurological deterioration in females with acute ischemic stroke. Front Neurol.

[16] Hacke W, Kaste M, Bluhmki E, Brozman M, Dávalos A, Guidetti D, Larrue V, Lees KR, Medeghri Z, Machnig T (2008). Thrombolysis with alteplase 3 to 4.5 hours after acute ischemic stroke. N Engl J Med.

[17] Tanaka R, Ueno Y, Miyamoto N, Yamashiro K, Tanaka Y, Shimura H, Hattori N, Urabe T (2013). Impact of diabetes and prediabetes on the short-term prognosis in patients with acute ischemic stroke. J Neurol Sci.

[18] Hong JM, Kim DS, Kim M (2021). Hemorrhagic transformation after ischemic stroke: mechanisms and management. Front Neurol.

[19] Seners P, Turc G, Tisserand M, Legrand L, Labeyrie MA, Calvet D, Meder JF, Mas JL, Oppenheim C, Baron JC (2014). Unexplained early neurological deterioration after intravenous thrombolysis: incidence, predictors, and associated factors. STROKE.

[20] Chung JW, Kim N, Kang J, Park SH, Kim WJ, Ko Y, Park JH, Lee JS, Lee J, Yang MH (2015). Blood pressure variability and the development of early neurological deterioration following acute ischemic stroke. J Hypertens.

[21] Park TH, Lee JK, Park MS, Park SS, Hong KS, Ryu WS, Kim DE, Park MS, Choi KH, Kim JT (2020). Neurologic deterioration in patients with acute ischemic stroke or transient ischemic attack. NEUROLOGY.

[22] Gong P, Zhou J, Zhang Y (2021). Letter by Gong et al regarding article, "predictors of unexplained early neurological deterioration after endovascular treatment for acute ischemic stroke". STROKE.

[23] Mori M, Naganuma M, Okada Y, Hasegawa Y, Shiokawa Y, Nakagawara J, Furui E, Kimura K, Yamagami H, Kario K (2012). Early neurological deterioration within 24 hours after intravenous rt-PA therapy for stroke patients: the stroke acute management with urgent risk factor assessment and improvement rt-PA registry. Cerebrovasc Dis.

[24] Wei XE, Zhao YW, Lu J, Li MH, Li WB, Zhou YJ, Li YH (2015). Timing of recanalization and outcome in ischemic-stroke patients treated with recombinant tissue plasminogen activator. Acta Radiol.

[25] Battey TW, Karki M, Singhal AB, Wu O, Sadaghiani S, Campbell BC, Davis SM, Donnan GA, Sheth KN, Kimberly WT (2014). Brain edema predicts outcome after nonlacunar ischemic stroke. STROKE.

[26] Jung S, Schindler K, Findling O, Mono ML, Fischer U, Gralla J, El-Koussy M, Weck A, Galimanis A, Brekenfeld C (2012). Adverse effect of early epileptic seizures in patients receiving endovascular therapy for acute stroke. STROKE.

[27] Cui Y, Meng WH, Chen HS (2022). Early neurological deterioration after intravenous thrombolysis of anterior vs posterior circulation stroke: a secondary analysis of INTRECIS. Sci Rep.

[28] Jickling GC, Liu D, Stamova B, Ander BP, Zhan X, Lu A, Sharp FR (2014). Hemorrhagic transformation after ischemic stroke in animals and humans. J Cereb Blood Flow Metab.

