# Correction: Early neurological deterioration in acute ischemic stroke patients after intravenous thrombolysis with alteplase predicts poor 3-month functional prognosis - data from the Thrombolysis Implementation and Monitor of Acute Ischemic Stroke in China (TIMS-China)

**DOI:** 10.1186/s12883-022-02865-1

**Published:** 2022-09-12

**Authors:** Fengli Che, Anxin Wang, Yi Ju, Yarong Ding, Honglian Duan, Xiaokun Geng, Xingquan Zhao, Yongjun Wang

**Affiliations:** 1 Present Address: Department of Neurology, Beijing Tiantan Hospital, Capital Medical University, Beijing, China; 2 Department of Neurology, Beijing Luhe Hospital, Capital Medical University, Beijing, China; 3 Tiantan Neuroimaging Center for Excellence, Beijing Tiantan Hospital, Capital Medical University, Beijing, China; 4 Research Unit of Artificial Intelligence in Cerebrovascular Disease, Chinese Academy of Medical Sciences, Beijing, China; 5 China National Clinical Research Center for Neurological Diseases, Advanced Innovation Center for Human Brain Protection, Beijing Tiantan Hospital, Capital Medical University, Beijing, China

**Correction: BMC Neurol 22, 212 (2022)**

**https://doi.org/10.1186/s12883-022-02737-8**

Following publication of the original article [1], the authors reported an error found in affiliations 1 and 2.

The updated affiliations are given below and the changes have been highlighted in bold.

1 Present Address: Department of Neurology*, ***Beijing Tiantan Hospital***,* Capital Medical University, Beijing, China.

2 Department of Neurology, **Beijing Luhe Hospital***,* Capital Medical University, Beijing, China.

The original article [1] has been updated.
