# Supplementary material for: Early neurological deterioration in acute ischemic stroke patients after intravenous thrombolysis with alteplase predicts poor 3-month functional prognosis - data from the Thrombolysis Implementation and Monitor of Acute Ischemic Stroke in China (TIMS-China)
Source: BMC Neurol. 2022 Jun 7;22:212. doi: 10.1186/s12883-022-02737-8 (PMC9172113; doi:10.1186/s12883-022-02737-8)
Supplement: Supplementary file 1 — Additional file 1. [file 12883_2022_2737_MOESM1_ESM.docx]

Non-standard Abbreviations and Acronyms

| TIMS-China | Thrombolysis Implementation and Monitor of Acute Ischemic Stroke in China |
| --- | --- |
| END | early neurological deterioration |
| rt-PA | recombinant tissue-type plasminogen activator |
| NIHSS | National Institute of Health Stroke |
| mRS | modified Rankin Scale |
| sICH | symptomatic intracranial hemorrhage |
| SITS-MOST | Stroke-Monitoring Study |
| NINDS | National Institute of Neurological Disorders and Stroke |
| ECASS Ⅱ | European Cooperative Acute Stroke Study Ⅱ |
| IQRs | interquartile ranges |
| ORs | odd ratios |
| CIs | confidence intervals |
| SBP | systolic blood pressure |
| DBP | diastolic blood pressure |
| OCSP | Oxfordshire Community Stroke Project |
| TIA | transient ischemic attack |
| TOAST | Trial of Org 10172 in Acute Stroke Treatment |
| WBC | white blood cell |
| PLT | platelet |
| INR | international normalized ratio |
| PT | prothrombin time |
| APTT | activated partial thromboplastin time |
| FBG | fibrinogen |
| LDL-C | low-density lipoprotein cholesterol |
| TC | cholesterol |
| CE | cardioembolism |
| LAA | large- artery atherosclerosis |
